# Retroperitoneal liposarcomas: the experience of a tertiary Asian center

**DOI:** 10.1186/1477-7819-9-12

**Published:** 2011-02-01

**Authors:** Ser Yee Lee, Brian Kim Poh Goh, Melissa Ching Ching Teo, Min Hoe Chew, Pierce Kah Hoe Chow, Wai Keong Wong, London LPJ Ooi, Khee Chee Soo

**Affiliations:** 1 Department of General Surgery, Singapore General Hospital, Outram Road, 169608, Singapore; 2 Department of Surgical Oncology, National Cancer Centre, 11 Hospital Drive,169610, Singapore; 3 Duke-NUS Graduate Medical School, 8 College Road, 169857, Singapore

## Abstract

**Background:**

Liposarcoma is the single most common soft tissue sarcoma in the retroperitoneum.

**Materials and methods:**

A retrospective review of patients with primary retroperitoneal liposarcoma treated between June 1990 and June 2005 were conducted to evaluate the clinical results of resection for retroperitoneal liposarcomas (RPLS) and the prognostic factors for disease recurrence and patient survival in an Asian population.

**Results:**

Twenty-one patients operated on for curative intent (12 Males, 9 Females; mean age: 52.4 years) were evaluated. Of these, 13 presented with tumors that were well differentiated (61.9%), 4 (19.0%) with myxoid/round cell, 3 (14.3%) with dedifferentiated and 1(4.8%) with pleomorphic morphology. The median tumor burden was 36 cm (9-83). Median follow-up time was 62 months. There was no peri-operative mortality and morbidity occurred in 6(28.6%) patients. Surgical margins were involved in 10(47.6%) patients. Resection of contiguous organs was required in 15(71.4%) to achieve gross surgical margins. Eleven out of the 21(52%) of the patients had recurrence of the tumor. Median disease-free survival was 19 months and the overall 3- and 5-year survival rate was 87% and 49% respectively.

**Conclusion:**

An aggressive surgical approach in both primary and recurrent RPLS in our institution is associated with 3- and 5-year survival rate of 87% and 49% respectively. Contiguous organ resection is often required to achieve local control.

## Introduction

Soft tissue sarcomas are rare and account for less than 1% of all newly diagnosed malignancies. One third of malignant tumors that arise in the retroperitoneum are sarcomas. Liposarcoma is the single most common soft tissue sarcoma and the most common retroperitoneal sarcoma. It accounts for at least 20% of all sarcomas in adults and up to 41% of all retroperitoneal sarcomas[1,2]. Retroperitoneal liposarcomas (RPLS) grow slowly and silently. Its prognosis is poor compared to the other histological subtypes of retroperitoneal sarcomas[3,4]. Only complete excision provides a hope of a cure, this is often difficult, especially in well differentiated subtypes because the margins are not grossly apparent thus often necessitating contiguous organ resection. Classification of liposarcoma into subtypes based on morphologic features and cytogenetic aberrations is now widely accepted. The 4 subtypes includes Well-differentiated, De-differentiated, Myxoid/Round cell and Pleomorphic[5].

Previous studies have shown that high histological grade and incomplete gross resection are the most important negative prognostic factors in patients with retroperitoneal sarcoma. Complete surgical excision is the mainstay of treatment. Some previous reports suggested that there is no survival benefit of partial resection as compared to biopsy alone without resection [2,6-8]. There is however no universal agreement and at least one series reported that in selected patients with retroperitoneal liposarcomas, partial resection can prolong survival and provide palliation[1]. medical therapies have shown some efficacy in the management of RPLS, although most consensus is that total surgical extirpation provides the patient best chance for cure[9-11].

The aim of this study is to review our experience in the management of RPLS in an Asian population and to identify any associated prognostic factors.

## Methods

Between July 1990 and June 2005, 91 consecutive patients who underwent surgical resection for a retroperitoneal tumor or mass at our institution were identified from a prospectively maintained database. Twenty-one patients with primary pathologically proven retroperitoneal liposarcoma were treated between this period. Their clinical data and operative notes, radiological reports and pathological reports were reviewed retrospectively. Histology at primary presentation was reviewed and classified into 4 distinct subtypes (Well-differentiated, De-differentiated, Myxoid/Round cell and Pleomorphic) according to the World Health Organization (WHO) classification and graded 1, 2 or 3 according to the French Federation of Cancer Centers Sarcoma Group grading systems[3,12,13].

Morbidity and mortality analyses were conducted by reviewing patient charts and clinical records. Operative morbidity and mortality was defined as any significant complications or death within 30 days of surgery following surgery. Significant complications included wound infection and dehiscence, reactionary hemorrhage necessitating repeat surgery, post-operative pneumonia, culture-proven septicemia, radiological identification of an intra-abdominal abscess, enterocutaneous fistula or confirmed deep vein thrombosis and/or pulmonary embolism. Margins were defined as microscopically clear if there was not tumor within 1 mm or more of the edge of the inked surgical margin. The tumor burden was determined by the sum of the 3 maximum tumor diameters and tumor size was defined as the maximum tumor diameter.

Patients were followed up at the specialist outpatient clinics at approximately 3-month intervals during the first year and 6-month intervals thereafter. Information obtained during follow-up included status of disease (alive with or without clinical evidence of disease, dead of disease or treatment, dead of other causes without evidence of disease). CT or MRI was performed at 6-month interval or earlier if there was clinical suspicion of recurrence or at the surgeon's discretion. Recurrence was defined as the time of initial surgery to confirmation of clinical recurrence by imaging e.g. CT or MRI.

In this study, the Kaplan-Meier estimate of the survival curve was used to summarize the data. Univariate analysis and comparison was performed for each factor of interest using Tarone-ware test. Tarone Ware test is a modification of the log rank test for comparing two survival curves with censored data and it is chosen as its key benefit is that it is designed to provide a valid statistical test, even with a large fraction of censored data. P < 0.05 was considered statistically significant[14].

## Results

### Clinico-pathological characteristics

The patient's demographic, surgical and pathological data are summarized in Table 1. Twenty-one patients with primary retroperitoneal liposarcoma operated on with curative intent (12 Males, 9 Females; mean age: 52.4 years, range: 29-71) were evaluated. The median age for patients at presentation was 53.13 years; most of them were above 40 years of age, except one who was 28 years old. The median duration of hospitalization was 10 days (range: 7 to 27 days). The most common symptom at presentation was abdominal discomfort and distension (24%) and 2 patients presented with symptoms as a result of mass effect namely bilateral lower limb edema and urinary frequency. An abdominal mass was palpable in the majority, 16 of the patients at presentation (76%). Of these, 13 presented with tumors that were well differentiated (61.9%), 4 (19.0%) with myxoid/round cell, 3 (14.3%) with dedifferentiated and 1(4.8%) with pleomorphic morphology. Eleven patients' (52.4%) tumors were classified as grade 1, 3 were grade 2(14.3%) tumors and the remaining 7 were grade 3(33.3%) tumors. The tumor burden was determined by the sum of the 3 maximum tumor diameters and tumor size was defined as the maximum tumor diameter. The median tumor burden was 36 cm (Range 9-83 cm). Twelve patients had tumors maximum diameter smaller than 20 cm whereas 9 patients has tumor larger then 20 cm. The largest tumor diameter was 43 cm. Median follow-up time was 62 months (Range 0.05 to 10.39 years). There was no post-operative mortality, morbidity occurred in 6(28.6%) patients. Surgical margins were involved in 10(47.6%) patients. Resection of contiguous organs was required in 15(71.4%) to achieve gross surgical margins.(Table 1) Eleven out of the 21(52%) of the patients had tumor recurrence.

**Table 1:** Clinico-pathologic and Treatment Characteristics in Patients with Primary Liposarcoma of the Retroperitoneum

Variables	Mean/median/n (percentage %)
Age	
Mean (std)	53.36 (11.47)
Median (range)	53.13 (28.56, 71.89)
Gender (n, %)	
Male	12 (57.1)
Female	9 (42.9)
Duration of Hospitalization	
Median (range)	10 (7, 27)
Grade (n, %)	
Grade 1	11 (52.4)
Grade 2	3 (14.3)
Grade 3	7 (33.3)
Histology (n, %)	
Well differentiated	13 (61.9)
Myxoid/Round cell	4 (19.0)
DeDifferentiated	3 (14.3)
Pleomorphic	1 (4.8)
Tumor size (n, %)	
< = 20 cm	12 (57.1)
> 20 cm	9 (42.9)
Margins (n, %)	
Positive	10 (47.6)
Negative	11 (52.4)
Resection of contiguous organs (n, %)	
Yes	15 (71.4)
No	6 (28.6)

### Disease free and overall survival analysis

**Figure 1:**
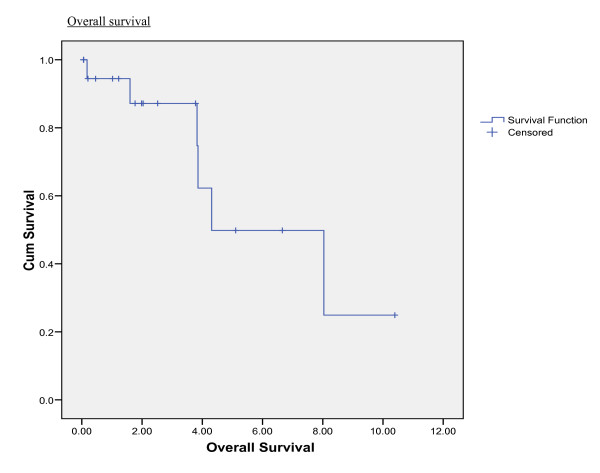


In this series, median disease-free interval and median overall survival was 19 months and 52 months respectively. The overall 3- and 5-year survival rate was 87% and 49% respectively. (Figure 1)

The univariate analysis of gender, age at presentation, tumor size, positive surgical margins, tumor differentiation, tumor grade and presence of contiguous organ resection were analyzed with regards to disease-free survival and overall 3- and 5-year survival rate. This was summarized in Table 2.

**Table 2:** Risk factors for overall survival and disease free survival after operation (p-values of the Tarone-ware test are presented)

Risk factors	3-year survival rate	5-year survival rate	p-value
Gender			0.544
Male	90%	67.5%	
Female	83.3%	27.8%	
Age (>50 years)			0.843
< = 50	83.3%	55.6%	
>50	90%	45%	
Tumor size (> 20 cm)			0.379
< = 20	100%	66.7%	
> 20	80.8%	40.4%	
Margins			0.757
Negative	87.5%	58.3%	
Positive	88.9%	44.4%	
Degree of Differentiation			0.997
Well differentiated	90.9%	83.3%	
Not well differentiated	54.5%	41.7%	
Grade			0.718
1	88.9%	44.4%	
2 & 3	85.7%	57.1%	
Organ resection			0.248
Yes	100%	40%	
No	60%	60%	

Tarone-ware test (which can be applied in case of non proportional hazard) was used to compare the overall survival rates for each covariates of interest. Although 3-year and 5-year survival rates were both higher for male patients, patients with tumor size >20 cm and well differentiated, no significant difference could be detected using Tarone-ware test, this might be due to the small sample size.

In our series, females have a 3- and 5-year overall survival (OS) at 83.3% and 27.8%. respectively; the males patients have a 3-year OS of 90% and 5-year OS of 67.5%, this was not statistically significant. In comparing the age of presentation, patients older than 50 years of age have a 90% and a 45% 3- and 5-year OS respectively, as compared to 83.3% and 55.6% for 3- and 5 year-OS in patients who are younger than 50 years of age. Three year DFS is at 33.3% for patients who presented at 50 years of age and 61% for those 50 years or older. This is not statistically significant.

Patients with tumors that are 20 cm or larger have a trend to do worse but no significance is detected. The 3-year; 5-year OS and 3-year DFS for patients whose tumors that are larger than 20 cm at presentation are 80.8%, 40.4% and 28.1% as compared to 100%, 66% and 75% respectively (Patients with largest tumor diameter smaller then 20 cm)(P = 0.379). Patients with positive margins also seem to have a worse trend in terms of overall survival and recurrence. Patients with positive microscopic margins had a 3-year OS, 5-year OS and 3-year DFS rate of 88.9%, 44.4% and 31.7% as compared to 87.5%, 58.3% and 62.5% respectively, in patients with negative microscopic margins.(P = 0.757). Patients with well differentiated tumors had a trend for a better 3-, 5-year OS than those with other subtypes (90.9% and 83.3% vs. 54.5% and 44.4%), however, the well differentiated tumors tend to recur earlier than the other subtypes in our series of patients (DFS, 32.8% vs. 60%), although there is no significance detected. This is consistent with the nature of well differentiated liposarcomas which is known to have more loco-regional recurrences than other subtypes.

We did not detect any statistical difference in OS and DFS between the different tumor grades Patients who required contiguous organ resection to achieve gross surgical resection also do not do worse in our study. The 3-year OS, 5-year OS and 3-year DFS for patients who required contiguous organ resection were 100%, 40% and 40% as compared to 60%, 60% and 51.4%(patients without the need for contiguous organ resection, respectively.(P = 0.248) (Table 2) In the group of patients with contiguous organ resection (n = 15), the kidney was the most common organ resected (n = 5), followed by the colon (n = 4), the spleen(n = 2) and the pancreas (n = 2). Four patients required 2 or more organs resected.

There was no significance prognostic factors detected in our series, this is probably due to small sample size with the result of a Type II error.

## Discussion

Liposarcomas is the most common mesenchymal tumor of the retroperitoneal space but RPLS continues to pose a challenge with regards to diagnosis, prediction of clinical behavior, and treatment of disease recurrence within the intra-abdominal and retroperitoneal space. Retroperitoneal liposarcomas tend to be of low to intermediate grade, while other sarcomas of other histologic types e.g. leiomyosarcomas in this location tend to be high grade[4,15,16]. Sarcomas are believed to arise *de novo*, spreading by direct, local extension or hematogenous routes, metastases at the time of initial presentation are uncommon. However, if metastatic, the lungs are the most common site of initial metastases.

The two largest series to date on RPLS were published in the Western population by Neuhaus et al. and Singer et al., however, there is little data in the current literature describing RPLS in the Asian population[4,16]. Histological grade was consistently reported to be the most important factor affecting survival rates for patients with liposarcomas[17]. In our series by comparing tumors that are grade 1 against grade 2 and 3 tumors, there was no statistical significance detected probably due to the limited sample size. The median tumor burden of 36 cm for our patients also appears to be larger than that reported in western literature[4].

There are two widely accepted grading systems internationally for soft tissue sarcomas, the National Cancer Institute (NCI) and the French Federation of Cancer Centres (FNCLCC) grading system. Both systems have proven to have prognostic value and share several features e.g. the emphasis on the histological type and the evaluation of the amount of necrosis. Neither of the systems is endorsed solely by the Association of Directors of Anatomic and Surgical Pathology or the WHO as yet[12,18]. To further aid in risk stratification and prognostication, nomograms are becoming a popular tool; Memorial Sloan-Kettering has designed and validated their nomogram for 12-year sarcoma-specific mortality utilizing seven histological types as considerations to calculate the probability of a patient reaching a designated clinical end-point[19,20]. Despite these problems, grading of sarcomas has been an important progress pathologists have contributed to the treatment of sarcomas. Grading identifies patients at highest risk of distant metastasis and aggressive tumor behavior, thus helps and guides oncologists in the management of these patients.

From the literature, the overall 5-year survival for well-differentiated subtypes is 90%, while 5-year survival for pleomorphic subtypes is only 30-50%. De-differentiated and myxoid/round cell subtypes have intermediate 5-year survival rates of 75% and 60-90%, respectively. Well-differentiated liposarcomas may recur locally, but metastatic potential is low. Pleomorphic liposarcomas have high metastatic potential, accounting for the decreased rate of survival[4]. It been reported recently that well differentiated liposarcomas and de-differentiated liposarcomas have different biological behaviors, in de-differentiated tumors, they tend to present as a recurrence more often, require multi-organ resection more frequently and has a shorter disease free interval when compared to well differentiated subtypes[21]. In our series, out of the 11 patients with recurrence, all of them had loco-regional recurrence and the majority of them have well differentiated subtypes (n = 7, 64%) with only one patient with concurrent liver metastases. Our aggressive surgical policy of achieving gross negative margins including contiguous organ resection if necessary have resulted in comparable survival rates despite the median tumor burden larger compared to the western literature.

In the literature, factors with negative prognostic value regarding survival include de-differentiation subtype, grade 2-3, stage II-III, size >20 cm, and involved surgical margins[4]. In our series, some of these factors also showed a negative prognostic trend although it did not reach any statistical significance. The retroperitoneal location is a negative prognostic factor and a significant risk factor when considering local recurrence of disease. Distant metastasis is more common with de-differentiation, grade II-III, and deep seated location[17]. Distant metastasis also relates to tumor size. In a review of 460 patients with liposarcoma of which 35% are RPLS (n = 159) who had achieved local control of their disease, recurrence and incidence of metastatic disease at 5 years was noted to increase significantly with increased tumor size at initial evaluation[22]. The RPLS is of special interest as the retroperitoneum is the second most common site of occurrence, with up to 36% of liposarcomas occurring at this site. The tumor is often deep-seated and large at the time of diagnosis as the retroperitoneum space provides a large potential volume allowing sizeable growth prior to development of signs and symptoms[4,17]. In our series, consistent with the current literature, we found that the presence of contiguous organ resection and tumor size of greater than 20 cm was negatively associated with prognosis, although due to a small sample size, there was no statistical significance detected. The mainstay of treatment is complete surgical resection. Complete resection was often challenging as the tumor may be difficult to distinguish from normal retroperitoneal fat. Furthermore, adjacent organs involved by the tumor may also need to be resected[4]. Failure to achieve macroscopic clearance was often due to the size of the tumor and the need for extensive visceral resection. Retroperitoneal liposarcomas is often large at presentation and can grow to enormous size, weighing over 100 pounds and measuring 50 cm in maximum diameter[23]. The largest tumor diameter in our series was 43 cm, with almost half of the tumors measuring more than 20 cm (42.9%). Studies had shown that complete resection may increase overall 5-year survival to 58% from 16.7%[17]. These tumors usually arise from the perinephric fat and as a result, kidney involvement was not unexpected, they often displaced the kidney peripherally or caused the kidney to be rotated away and in advanced cases the tumor may encase the kidney or cause pelvi-ureteric obstruction. Kidney was the most common organ resected followed by the colon and this was shown in our series as well. In a palliative setting, the colon was the organ most commonly resected followed by the kidney[16]. Notably, our series has a high rate of contiguous organ resection as compared to some earlier larger western series [4] (76% vs. 26%), this is mostly likely attributed by the larger tumor burden of our patients but our percentage of achieving negative microscopic margins is comparable to centres that advocate extensive resections[24,25]. We postulate that this is in part due to the later presentation of our Asian patients to tertiary healthcare[26,27]. This delay in presentation may be contributed by the cultural preference of our patients to seek traditional medical care over western medicine and the general reluctance of patients to obtain early medical attention for their symptoms, tending to ignore even significant symptoms till the disease is incapacitating or when family members coax and brings the patient to see a doctor The level of general medical knowledge is also poorer in the older generation in many Asian societies as compared to their Western counterparts. However in the recent years, as we understand that positive margins were associated with decreased survival, extensive or wide resection e.g. compartmental resections have been shown and advocated, to be performed especially in high volume tertiary centres, to achieve better outcomes[24,25].

There is no strong evidence that chemotherapy or radiotherapy is curative[28,29]. There is no prospective randomized controlled trial confirming the potential benefit of radiotherapy that emerges from retrospective studies[11,30]. Given the large size and truncal location of retroperitoneal liposarcomas, adjuvant radiation is often not an option secondary to substantial morbidity associated with the required radiation doses and fields. Similarly, in well-differentiated low grade tumors, adjuvant chemotherapy yields little benefit. In high-grade disease, administration of adriamycin and ifosfamide may yield partial responses in up to 50% of patients with increased overall survival; however, complete responses are seen in less than 10% of patients[11]. To date, there are few prospective clinical trials analyzing chemo-radiotherapy regimes for retroperitoneal sarcomas, there is none specific and solely for retroperitoneal liposarcoma histological subtype[11,30]. Retroperitoneal recurrences are often difficult to control, with death most often occurring from local effects of the tumor burden[17].. Despite an aggressive surgical approach, in our series we only achieve 5 year OS of 49%, this is probably in part attributed to the poor efficacy of neoadjuvant and adjuvant therapies. As such, the biology and molecular alteration of this disease need to be further characterized with more basic and translational research to explore new, innovative targeted therapeutic agents that target specific translocation or amplification products, this approach forward may offer promise for this rare and lethal disease[31].

As RPLS is a rare entity, a multi-institution prospective database will serve well to understand this disease more comprehensively. The limitations of our study is that the review is retrospective in nature and thus prone to bias, due to the limited sample size, there will be a element of type II error, resulting in a difficulty of achieving firm conclusion and statistical significance with regards to the prognostic factors analysis.

## Conclusion

The experience in our institution represents the surgical experience and behavior of RPLS in an Asian Population and we have demonstrated that the behavior of RPLS and most of the results seemed consistent with the current western literature although our patients' tumor burden appears to be slightly larger than that reported in western literature, necessitating a larger percentage of them undergoing contiguous organ resection to achieve gross clear margins. We believe that complete surgical resection is the most important component of treatment even if it necessitates multiple organ resections to achieve gross surgical margins, as it improves survival.

## Competing interests

The authors declare that they have no competing interests.

## Authors' contributions

SYL designed, coordinated the study, carried out the extraction of data, performed critical appraisal of the literature and wrote the manuscript. MHC coordinated the project, assisted in review and collection of the clinical data and assisted in writing the manuscript. BKPG developed the literature search, carried out the extraction of data and critically reviewed the manuscript. MT, PKHC, WKW, LLO, KCS supervised, assisted in the critical appraisal of included studies and critically reviewed the manuscript. All authors contributed significantly to this work, read and approved the final manuscript.
